# Artificial intelligence in Brazilian Primary Health Care: scoping review

**DOI:** 10.1590/0034-7167-2024-0363

**Published:** 2025-12-08

**Authors:** Dany Alexandra Morales Botero, Rafaele Bonfim, Karina Fonseca, Rubia Laine de Paula Andrade-Gonçalves, Aline Aparecida Monroe, Fátima Morales

**Affiliations:** I University of Sevilla. Sevilla, Andalucia, Spain; II Universidade de São Paulo. Ribeirão Preto, São Paulo, Brazil

**Keywords:** Artificial Intelligence, Primary Health Care, Health Systems, Brazil, Review., Inteligência Artificial, Atenção Primária à, Saúde, Sistemas de Saúde, Brasil, Revisão., Inteligencia Artificial, Atención Primaria de Salud, Sistemas de Salud, Brasil, Revisión.

## Abstract

**Objectives:**

to provide evidence on the usage of artificial intelligence in Brazilian Primary Health Care.

**Methods:**

scoping review, conducted on February 2025. Searches were accomplished in six databases. Selection process was conducted by three reviewers. Data were extracted using a form by two reviewers. Results were synthetized in a narrative way.

**Results:**

27 studies were included out of 981 retrieved. Machine Learning and Deep Learning were used in the studies to: make estimates and predictions, track and diagnose health conditions and complications and analyze determinants of universal health coverage. The main strengths found for using them were: to optimize resources; to provide early diagnosis; to allow risk stratification; to facilitates access. The main challenges for using them were: poor access to treatment and data availability.

**Conclusions:**

although the strengths of AI applications in Brazilian healthcare system were highlighted, its implementation should address the guarantee of the effective right to health.

## RESUMO

**Objetivos:**

fornecer evidências sobre o uso de inteligência artificial na Atenção Primária à Saúde no Brasil.

**Métodos:**

revisão de escopo, conduzida em fevereiro de 2025. As buscas foram realizadas em seis bases de dados. O processo de seleção foi conduzido por três revisores. Os dados foram extraídos por meio de um formulário por dois revisores. Os resultados foram sintetizados de forma narrativa.

**Resultados:**

981 estudos foram recuperados, dos quais, 27 foram incluídos. Aprendizado de Máquina e Aprendizado Profundo foram utilizados nos estudos para: fazer estimativas e previsões, rastrear e diagnosticar condições de saúde e complicações bem como analisar determinantes da cobertura universal de saúde. Os principais pontos fortes encontrados para seu uso foram: otimizar recursos; fornecer diagnóstico precoce; permitir estratificação de risco; facilitar o acesso. Os principais desafios para seu uso foram: acesso precário ao tratamento e disponibilidade de dados.

**Conclusões:**

embora os pontos fortes das aplicações de IA no sistema de saúde brasileiro tenham sido destacados, sua implementação deve abordar a garantia do direito efetivo à saúde.

## RESUMEN

**Objetivos:**

aportar evidencias sobre el uso de inteligencia artificial en la Atención Primaria de Salud en Brasil.

**Métodos:**

revisión de alcance realizada en febrero de 2025. Las búsquedas se efectuaron en seis bases de datos. La selección fue conducida por tres revisores. Los datos fueron extraídos mediante un formulario por dos revisores. Los resultados se sintetizaron de forma narrativa.

**Resultados:**

se recuperaron 981 estudios, de los cuales 27 fueron incluidos. El aprendizaje automático y el aprendizaje profundo se emplearon para: realizar estimaciones y predicciones; efectuar tamizaje y diagnóstico de condiciones de salud y complicaciones; y analizar determinantes de la cobertura sanitaria universal. Los principales puntos fuertes identificados fueron: optimizar recursos; proporcionar diagnóstico precoz; permitir la estratificación del riesgo; y facilitar el acceso. Los principales desafíos para su uso fueron: acceso precario al tratamiento y disponibilidad de datos.

**Conclusiones:**

aunque se destacaron los puntos fuertes de las aplicaciones de IA en el sistema de salud brasileño, su implementación debe contemplar la garantía del derecho efectivo a la salud.

## INTRODUCTION

In order to effectively guarantee health as a universal right, the integration of new technologies in the sector has been particularly important^(1)^. Although it is evident that the implementation of technologies such as artificial intelligence (AI) can guide the care management, it almost never addresses all people’s needs since they are countless, multidimensional and interdependent^(2)^.

In Brazil, Primary Health Care (PHC), as fundamental pillars of health systems^(3)^, focuses on facilitating access and continuity of health services, serving as the gateway to the country’s healthcare network. Since the promulgation of Decree 7,508 in 2011, a law that regulates the development of the Unified Health System, it explicitly recognizes that the function of PHC is to identify risks, needs, and demands of the population that can be addressed through qualified professionals, quality service, implementation of new technologies, research, public participation, and financing^(4)^.

This decree reinforced the recommendations of the National Policies on PHC, enhancing its role and supporting the implementation of the principles and directives of the Unified Health System. These policies enable significant transformations, particularly in the care model and the management of the health workforce in cities in Brazil^(5-7)^.

Therefore, in compliance with health regulations, the implementation of new technologies, such as AI, can anticipate improvements in access to health services, quality of care, and continuous innovation. This, in turn, enhances the sector’s development, especially when applied to PHC. Additionally, it is estimated that the use of it can contribute to much more sustainable, efficient, and adaptable health systems, tailored to the population’s needs, thereby it can help to promote equitable and universal access to health services^(8)^.

Since 1854, ideas like those of George Boole regarding AI have been emerging, arguing that it was possible to systematize logical reasoning in the same way mathematical equations are solved. However, it wasn’t until 1936, with the design of the Turing machine, that the initial theoretical model of AI began to be considered, allowing computers to perform rapid and powerful calculations and processing. Nevertheless, the most effective version did not emerge until the 1950s^(9)^.

According to the European Union’s AI Act, artificial intelligence is defined as software developed using one or more techniques and strategies capable of generating outputs such as content, predictions, recommendations, or decisions that influence the environments with which it interacts^(10)^. However, a recent scoping review indicates that research on AI in Primary Health Care remains in its early stages, underscoring the need for increased interdisciplinary collaboration and comprehensive evaluation studies^(11)^.

AI can be understood from three main perspectives. First, machine learning refers to the development of computer programs that learn from data through a training process, enabling them to identify patterns and make predictions on new, unseen data. During this process, input and output data are used to train an algorithm, which incrementally refines and optimizes its internal rules based on the data^(12)^.

In contrast, deep learning enables machines to recognize complex patterns in data, such as visual elements in images. This approach employs multiple layers of processing-resembling neural networks in the human brain-to identify structures and features within large datasets^(13)^. The initial layers typically focus on basic elements, while subsequent layers build upon previously acquired knowledge, progressively increasing the complexity and abstraction of the learned concepts^(14)^.

Finally, natural language processing (NLP) is a specialized integration of classical machine learning, deep learning, and computational linguistics. It allows computers to comprehend and process human language in both spoken and written forms. One of the most widely used models in NLP is the neural network, which simulates the biological functions of human neurons. These networks perform mathematical operations to process and share information in a way that mirrors the functioning of neurons in the brain^(15)^.

Given the evolving nature of AI applications in PHC, comprehensive studies are necessary to synthesize existing evidence on how AI is enhancing health care delivery, as well as to identify the challenges associated with its implementation.

## OBJECTIVES

To provide evidence on the types, applications, challenges, and strengths of artificial intelligence implementation in Brazilian PHC.

## METHODS

This study is a scoping review, with its protocol registered in the OSF Registries (https://doi.org/10.17605/OSF.IO/Q6ZMX). The review was conducted following the methodology outlined in the Joanna Briggs Institute Reviewer’s Manual for Scoping Reviews^(16)^ and the recommendations of the PRISMA-ScR guidelines^(17)^. A scoping review aims to map the existing literature on a specific topic by identifying the types of available evidence, clarifying key concepts and definitions, analyzing how research has been conducted in the field, identifying relevant characteristics or factors, and uncovering knowledge gaps^(18)^.

This scoping review followed the established stages: (1) identifying the research question; (2) identifying relevant literature; (3) selecting eligible publications; (4) extracting data; and (5) analyzing and synthesizing the evidence^(19,20)^.

To identify relevant publications, the research question-”How has artificial intelligence been applied in primary health care in Brazil?”-was developed using the PCC framework, in which: P (Population) = Primary Health Care (PHC); C (Concept) = Application of Artificial Intelligence (AI); and C (Context) = Brazil.

Eligible sources included journal articles and research abstracts, as well as experimental, quasi-experimental, and observational studies. Additionally, the review considered studies on diagnostic accuracy, intervention protocols, literature reviews, descriptive and qualitative studies, opinion pieces, commentaries, and editorials.

Studies were included if they addressed the types of AI being used and how AI has been applied in PHC settings in Brazil. The following databases were searched: Embase, Scopus, MEDLINE, LILACS, Web of Science, and Epistemonikos. A search strategy was developed using Health Sciences Descriptors (DeCS/MeSH) relevant to the topic (Chart 1), and adapted for each individual database or source.

**Chart 1:** Article search strategies conducted for the scoping review on artificial intelligence application in Primary Health Care in Brazil according to the consulted databases, Ribeirão Preto, São Paulo, Brazil, 2025

Database	Search Strategies
**Embase**	#1‘artificial intelligence’/exp OR ‘artificial intelligence’ OR ‘artificial-intelligence’/exp OR ‘artificial-intelligence’ OR ‘machine learning’/exp OR ‘machine learning’ OR ‘machine intelligence’/exp OR ‘machine intelligence’ OR ‘deep learning’/exp OR ‘deep learning’ #2‘primary care’/exp OR ‘primary care’ OR ‘primary health care’/exp OR ‘primary health care’ OR ‘primary healthcare’/exp OR ‘primary healthcare’ OR ‘primary attention’ OR ‘primary-care’/exp OR ‘primary-care’ OR ‘primary-health-care’/exp OR ‘primary-health-care’ OR ‘primary-health care’/exp OR ‘primary-health care’ OR ‘primary health-care’/exp OR ‘primary health-care’ OR ‘primary-healthcare’/exp OR ‘primary-healthcare’ OR ‘primary-attention’ #3‘brazil’/exp OR brazil OR ‘brazilian’/exp OR Brazilian #4#1 AND #2 AND #3
**Scopus**	TITLE-ABS-KEY (“artificial intelligence” OR “artificial-intelligence” OR “machine learning” OR “machine intelligence” OR “deep learning” OR “big data” OR “digital data” OR “neural network”) AND TITLE-ABS-KEY (“primary care” OR “primary health care” OR “primary healthcare” or “primary attention” OR “primary-care” OR “primary-health-care” OR “primary-health care” OR “primary health-care” OR “primary-healthcare” or “primary-attention”) AND TITLE-ABS-KEY (Brazil OR Brazilian)
**MEDLINE**	(“artificial-intelligence”[All Fields] OR “artificial-intelligence”[All Fields] OR “machine learning”[All Fields] OR “machine intelligence”[All Fields] OR “deep learning”[All Fields] OR “big data”[All Fields] OR “digital data”[All Fields] OR “neural network”[All Fields]) AND (“primary-care”[All Fields] OR “primary health care”[All Fields] OR “primary-healthcare”[All Fields] OR “primary-attention”[All Fields] OR “primary-care”[All Fields] OR “primary health care”[All Fields] OR “primary health care”[All Fields] OR “primary health care”[All Fields] OR “primary-healthcare”[All Fields] OR “primary-attention”[All Fields]) AND (“brazil”[MeSH Terms] OR “brazil”[All Fields] OR “brazil s”[All Fields] OR “brazils”[All Fields] OR (“brazilian people”[Supplementary Concept] OR “brazilian people”[All Fields] OR “brazilian”[All Fields] OR “brazilians”[All Fields]))
LILACS^*^	(“artificial intelligence” OR “artificial-intelligence” OR “machine learning” OR “machine intelligence” OR “deep learning” OR “big data” OR “digital data” OR “neural network” OR “*inteligencia artificial*” OR *redes neurais* OR “*internet de las cosas*” OR “*maquinas de aprendizaje*” OR “*maquinas inteligentes*” OR “*redes neuronales*” OR “*datos digitales*”) AND (“primary care” OR “primary health care” OR “primary healthcare” or “primary attention” OR “primary-care” OR “primary-health-care” OR “primary-health care” OR “primary health-care” OR “primary-healthcare” or “primary-attention” OR “*atenção primária à saúde*” OR “*atenção primária*” OR “*atenção básica*” OR “*atención primaria de la salud*” OR “*atención primaria*” OR “*atención básica*”) AND (Brazil OR Brazilian OR *Brasil* OR *brasileira* OR *brasileña*)
**Web of Science**	((TS=(“artificial intelligence” OR “artificial-intelligence” OR “machine learning” OR “machine intelligence” OR “deep learning” OR “big data” OR “digital data” OR “neural network”)) AND TS=(“primary care” OR “primary health care” OR “primary healthcare” or “primary attention” OR “primary-care” OR “primary-health-care” OR “primary-health care” OR “primary health-care” OR “primary-healthcare” or “primary-attention”)) AND TS=(Brazil OR Brazilian)
**Epistemonikos**	(title:(“artificial intelligence” OR “artificial-intelligence” OR “machine learning” OR “machine intelligence” OR “deep learning” OR “big data” OR “digital data” OR “neural network” OR “*inteligencia artificial*” OR *redes neurais* OR “*internet de las cosas*” OR “*maquinas de aprendizaje*” OR “*maquinas inteligentes*” OR “*redes neuronales*” OR “*datos digitales*”) OR abstract:(“artificial intelligence” OR “artificial-intelligence” OR “machine learning” OR “machine intelligence” OR “deep learning” OR “big data” OR “digital data” OR “neural network” OR “*inteligencia artificial*” OR *redes neurais* OR “*internet de las cosas*” OR “*maquinas de aprendizaje*” OR “*maquinas inteligentes*” OR “*redes neuronales*” OR “*datos digitales*”)) AND (title:(“primary care” OR “primary health care” OR “primary healthcare” OR “primary attention” OR “primary-care” OR “primary-health-care” OR “primary-health care” OR “primary health-care” OR “primary-healthcare” OR “primary-attention” OR “*atenção primária à saúde*” OR “*atenção primária*” OR “*atenção básica*” OR “*atención primaria de la salud*” OR “*atención primaria*” OR “*atención básica*”) OR abstract:(“primary care” OR “primary health care” OR “primary healthcare” OR “primary attention” OR “primary-care” OR “primary-health-care” OR “primary-health care” OR “primary health-care” OR “primary-healthcare” OR “primary-attention” OR “*atenção primária à saúde*” OR “*atenção primária*” OR “*atenção básica*” OR “*atención primaria de la salud*” OR “*atención primaria*” OR “*atención básica*”)) AND (title:(Brazil OR Brazilian OR *Brasil* OR *brasileira* OR *brasileña*) OR abstract:(Brazil OR Brazilian OR *Brasil* OR *brasileira* OR *brasileña*))

* *In the Virtual Health Library (Biblioteca Virtual da Saúde), words were searched in the title, abstract, and subject, and the LILACS filter was used in the database consulted.*

The search was conducted by a single researcher in February 2025, using the identified keywords and Boolean operators AND and OR. Specifically, the OR operator was used between synonyms or related terms (e.g., “term1” OR “term2”), while AND was used to combine different conceptual components of the search query (e.g., “concept A” AND “concept B” AND “concept C”). The search was limited to studies published in Portuguese, English, and Spanish.

After the literature search, all identified citations were exported to the Rayyan QCRI online systematic review application of the Qatar Computing Research Institute^(21)^. After export, duplicate publications were excluded, and the remaining studies were submitted to a selection process through the reading of their abstracts and titles by two independent reviewers. Any disagreements that arise between the reviewers in the selection process was resolved by an additional reviewer. Eligible publications were read fully to a second selection process. The reasons for the exclusion of these articles were reported in the study. The process for including studies in the review were presented in a flowchart, as proposed by the 2020 Statement of Preferred Reporting Items for Systematic Reviews and Meta-Analyses (PRISMA)^(22)^.

Data were extracted from the articles included in the scoping review by one reviewer and verified by another reviewer using a data extraction form, constituted by the items: authors, year of publication, journal name, type of publication, language, study objective, study design, study location, study population/sample, types of AI used in PHC in Brazil, applications of the use of AI in PHC in Brazil, strengths faced by PHC in Brazil to implement AI and challenges faced by PHC in Brazil to implement AI.

The evidence presented directly answers the review’s objective and the guiding question. As recommended by Peters et al. (2020)^(16)^, the presentation of results was synthesized in narrative form, which map or describe the results found on the topic studied.

## RESULTS

**Figure 1:**
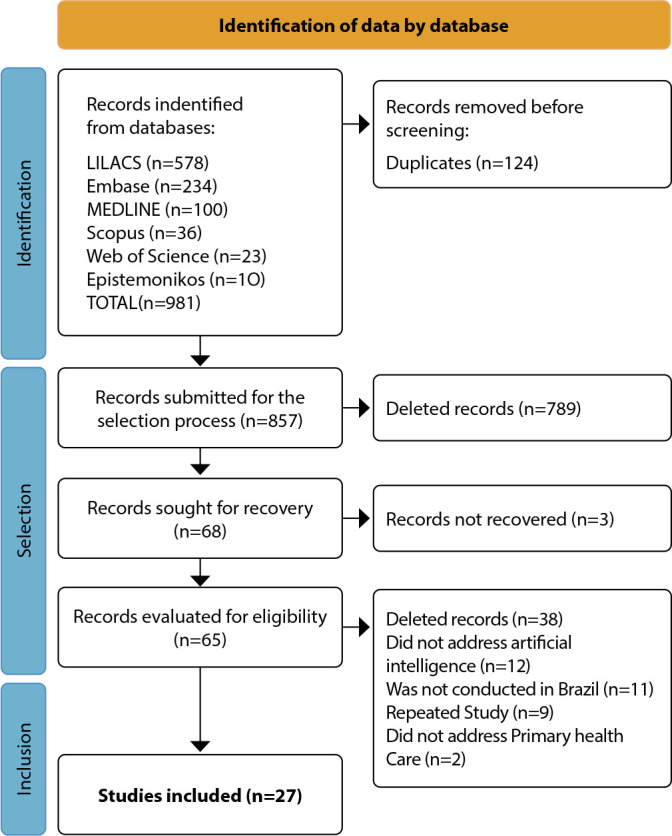
Flowchart of publications selection to the scoping review on artificial intelligence application in Primary Health Care in Brazil, 2025

*Source: Adapted from Page et al., 2021^(22)^.*

The search in the databases allowed the identification of 981 studies, of which 124 were excluded for being duplicates and 789 after reading their titles and abstracts. Thus, 68 publications were considered eligible for full reading, of which 27 were included in the review (Figure 1).

Among the 27 included publications, one (3.7%)^(23)^ was published in 2018, two (7.4%)^(24,25)^ in 2020, eight (29.6%)^(26-33)^ in 2021, two (7.4%)^(34,35)^ in 2022, six (22.2%)^(36-41)^ in 2023 and eight (29.6%)^(42-49)^ in 2024. The majority of publications (85.2%)^(23,25-28,30-36,38,39,41-49)^ were journal articles, and four (14.8%)^(24,29,37,40)^ correspond to abstracts of research results presented on conferences. Regarding the study designs, seven (25.9%)^(23,24,30,33-35,37)^ were retrospective studies, three (11.1%)^(27,36,46)^ were studies of diagnostic accuracy, three (11.1%)^(39,45,47)^ were descriptive studies, three (11.1%)^(26,38,48)^ were cross-sectional studies and three (11.1%)^(40,41,44)^ a prediction model. The study objectives were heterogeneous, being that some^(23,24,28,30,35,38,40-42,44,45,48)^ aimed to predict some health conditions or outcomes using AI, some^(25-27,32,33,36,37,46,47,49)^ evaluated their diagnostic accuracy, one^(29)^ proposed to assess the use of AI in decision making and in PHC monitoring, one^(31)^ to estimate resources needed to an immunization campaign implementation, one^(34)^ to predict the universal health coverage, one^(39)^ to assess the elderly people and one^(43)^ to analyze AI recommendations. The population of the studies included in this review can be seen in Chart 2.

**Chart 2:** Description of articles included in the scoping review on artificial intelligence application in Primary Health Care in Brazil, 2025

Authors, year of publication / Journal Name	Type of publication	Study design	Objective	Study population
Chiavegatto Filho et al., 2018^(23)^ / Journal of Epidemiology	Journal article	Retrospective study	To predict life expectancy with machine learning algorithms	3,052 municipalities with more than 10,000 inhabitants
Mistro et al., 2020^(24)^ / Diabetes	Abstract of research results presented on a conference	Retrospective study	To develop a predictive model for the glycemic level of people with type 2 diabetes mellitus based on electronic health record data in PHC	Data on medical records in PHC
Sobrinho et al., 2020^(25)^ / IEEE, Instituto de Ingenieros Eléctricos y Electrónicos	Journal article	Systematic review within a machine learning experiment	To analyze the usage of machine learning to assist in the early diagnosis of Chronic Kidney Disease developing countries	Developing countries, including Brazil
Brito et al., 2021^(26)^/ PLOS Neglected Tropical Diseases	Journal article	Cross-sectional study	To analyze the ability of AI using electrocardiograms to diagnose left ventricular systolic dysfunction in patients with Chagas disease	1,304 patients
Giavina-Bianchi et al., 2021^(27)^ / PLoS One	Journal article	Diagnostic accuracy study	To develop an efficient computer-aided diagnosis system with machine learning to be used by physicians in the PHC centers in Brazil to diagnose skin cancers	Patients 18 years old with one or more suspicious malignant lesions
Lima et al., 2021^(28)^/ Nature Communications	Journal article	Prospective cohort	To assess if the difference between predicted electrocardiogram-age and chronological age in risk estimation of overall mortality using AI	218,169 patients from CODE-15% Study; 1,631 from SaMi-Trop Study; and 14,263 from the ELSA-Brazil study
Martins et al., 2021^(29)^ / International Journal of Stroke	Abstract of research results presented on a conference	Intervention protocol	To present a strategy of modifying the primary prevention for stroke in Brazil with the restructuring of PHC - one strategy remains on the implementation of decision, data collection and monitoring software enriched with AI	8 PHC Units to implement a pilot study
Rocha et al., 2021^(30)^ / The Lancet Regional Health	Journal article	Retrospective study	To pilot the development of a Machine Learning approach to predict the week of delivery based on minimal patient data	3,876,666 mothers with live births distributed across 3,929 Brazilian municipalities
Rocha et al., 2021^(31)^ / Ciência y Saúde Coletiva	Journal article	Ecological study	To analyze the use of spatial AI to estimate the resources needed to implement Brazil’s covid-19 immunization campaign	PHC centers
Souza Filho et al., 2021^(32)^ / Journal of Psychiatric Research	Journal article	Randomized clinical trial	To evaluate the performance of Machine Learning algorithms in the detection of depression in patients	971 patients followed up in Rio de Janeiro PHC
Malerbi et al., 2021^(33)^ / Journal of Medical System	Journal article	Retrospective study	To assess the tomographic presence of diabetic macular edema in type 2 diabetes mellitus patients screened for diabetic retinopathy with color fundus photographs and an AI algorithm	366 individuals with a previous diagnosis of type 2 diabetes mellitus
Kumar et al., 2022^(34)^ / Frontiers in Artificial Intelligence	Journal article	Retrospective study	To determine whether health expenditure indicators drive universal health coverage, and to analyze the importance of key determinants and their interactions with universal health coverage in three economic blocks using machine learning	GCC (Gulf Cooperation Council); BRICS (Brazil, Russia, India, China, and South Africa) and AUKUS (Australia, UK, and USA).
Araújo et al., 2022^(35)^ / International Journal of Medical Informatics	Journal article	Retrospective study	To predict the risk of covid-19 death through machine learning	6,979 patients with covid-19 confirmed
Jidling et al., 2023^(36)^ / PLOS Neglected Tropical Diseases	Journal article	Diagnostic accuracy study	To investigate whether a deep neural network can detect Chagas Disease and chronic Chagas cardiomyopathy from ECG tracings	1,556,767 patients from CODE Study; 2,054 from SaMi-Trop Study; 631 from REDS-II Study and 13,739 from the ELSA-Brazil study
Malerbi et al., 2023^(37)^ / Investigative Ophthalmology & Visual Science	Abstract of research results presented on a conference	Retrospective study	To assess the gradeability of retinal imaging obtained with a non-mydriatic handheld retinal camera and AI	968 people with diabetes mellitus followed at the PHC level
Szlejf et al., 2023^(38)^ / Revista Brasileira de Pesquisa Médica e Biológica	Journal article	Cross-sectional study	To develop and test machine learning models to predict cognitive impairment using variables obtained in PHC settings	8,291 participants of the baseline assessment of the ELSA-Brazil study
Siqueira et al., 2023^(39)^/ Cogitare Enfermagem	Journal article	Descriptive study	To describe the modeling of an Expert System for the Multidimensional Assessment of elderly people	Nursing specialists and Computer Science professionals
Sau et al.,2023^(40)^/ Heart	Abstract of research results presented on a conference	Prediction model	To explore the prognostic significance and important phenotypic and genotypic associations of Neural Networkderived ECG features in long-term mortality	1,558,421 ECGs recorded in PHC in Brazil
Koch Nogueira et at., 2023^(41)^/ European Journal of Pediatrics	Journal article	Prediction model	To reveal the signs and symptoms for the classification of pediatric patients at risk for chronic kidney disease using decision trees and extreme gradient boost models for predicting outcomes	376 children with chronic kidney disease and 376 healthy controls
Sau et al., 2024a^(42)^/European Heart Journal	Journal article	Prospective cohort study	To predict time to mortality, through two image-driven AI- ECG	1,163,401 ECGs from 189.539 people from three cohort studies
Sandreschi et al., 2024^(43)^/ Revista Pesquisa Qualitativa	Journal article	Methodological study	To analyze the responses of ChatGPT when questioned about physical activity recommendations for health based on the characteristics of users served by Brazilian PHC	Five researchers in the field of Physical Education
Bomfim, 2024^(44)^/ BMC Oral Health	Journal article	Prediction model	To predict adolescents with untreated dental caries through a machine-learning	615 adolescents approach using three different algorithms.
Ramos et al., 2024^(45)^/ JMIR Public Health and Surveillance	Journal article	Descriptive study	To present the design principles of the architecture for an early-alert surveillance system for anticipating outbreaks of pandemic potential	PHC
Reis et al, 2024^(46)^/ Diabetology & Metabolic Syndrome	Journal article	Diagnostic accuracy study	To train and evaluate the diagnostic accuracy of a machine learning algorithm for automated detection of diabetic retinopathy	15,816 images (4,590 patients) recorded by color fundus photographs
Melo et at., 2024^(47)^/ Diabetes Epidemiology and Management	Journal article	Descriptive study	To report the implementation of a diabetic retinopathy screening program, emphasizing the challenges and premature termination	PHC Centers in a Brazilian City
Costa Júnior et al., 2024^(48)^/ Journal of Clinical Medicine	Journal article	Cross-sectional study	To predict metabolic Syndrome in adolescents using artificial neural networks on non- invasive variables	2,064 adolescents aged 18 and 19 years old
Georgescu et at., 2024^(49)^/ JMIR Formative Research	Journal article	Prospective cohort	To illustrate the preliminary transferability of two established AI models designed to detect high depression and anxiety symptom scores.	69 Brazilian participants between 51 and 92 years old

*AI - artificial intelligence; ECG - electrocardiogram; PHC - Primary Health Care.*

In 14 studies^(24-28,31,33,36,37,39,42,46-48)^ addressing the research question, machine learning was used. Deep learning was employed by 13 other studies^(23,29,30,32,34,35,38,40,41,43-45,49)^. Natural language processing did not demonstrate applicability results.

Under the parameter of “Applicability”, 19 different variants were observed in which AI was used as cutting-edge technology in PHC. Furthermore, strengths and challenges associated with AI usage in PHC in Brazil are detailed in Chart 3.

**Chart 3:** Types, Applicability, Strengths, and Challenges of the application of artificial intelligence in Brazilian Primary Health Care, 2025

Types of artificial intelligence	Machine Learning^(23,29,30,32,34,35,38,40,41,43-45,49)^ Deep Learning^(24-28,31,33,36,37,39,42,46-48)^
**Applicability**	Life expectancy estimation^(23)^ Prediction of glycemic level im people with diabetes mellitus^(24)^ Diagnosis of Chronic Kedney Disease^(25,41)^ Chagas detection and its complications on electrocardiogram^(26,36)^ Skin cancer diagnosis^(27)^ Prediction of mortality by electrocardiogram^(28,40,42)^ Restructuraction to prevent stroke^(29)^ Prediction of week of birth delivery^(30)^ Estimate the resources needed in a covid-19 vaccination plan^(31)^ Mental disorder screening^(32,49)^ Diagnosis of ocular pathologies due to diabetes mellitus^(33,37,46,47)^ Analyse the determinants of universal health coverage^(34)^ Prediction risk of death from covid-19^(35)^ Diagnosis of cognitive impairment^(38)^ Identification of risk factors for elderly people^(39)^ Physical activity recommendations through ChatGPT^(43)^ Prediction of dental caries in adolescents^(44)^ Anticipation of potential pandemic outbreaks^(45)^ Prediction of metabolic syndrome^(48)^
**Strenghs**	Provides new knowledge in the field of health^(23)^ Improve service delivery^(23)^ Identify the effectiveness of treatments to optimize resources^(23,24,26,30,33)^ Automatic and economical^(23)^ Optimal performance tool in early diagnosis^(24,26,27,33,36,38,46)^ Diagnosis coincident with that of a specialist doctor^(25)^ Easy to interpret results^(25)^ Allows early risk stratification and mortality prediction^(28,32,35,39,41,42,48)^ Allows prevention of stroke^(29)^ Good connectivity to mobile data networks for vaccination registration^(31)^ Disease prediction, consumption prediction in Primary Health Care^(32,43,45)^ Faciliattes access in low-income areas^(33,34,37)^
**Challenges**	Acess to treatment^(25,35)^ Improve data availability^(25,35)^ Low availability of search variables (algorithms) ^(29,31,34,37)^ Low internet connection^(33)^ Ignorance of the disease^(37)^ Long distance for Primary Health Care access^(37)^ High implementation cost^(42,45)^ Incorporation of new technologies^(39)^ Control professional^(43)^ Organizational problems in the Primary Health Care^(47)^

## DISCUSSION

In the context of PHC, it was observed that, unlike machine learning and deep learning, NLP has not been widely utilized. This absence raises the hypothesis that NLP’s capacity to interpret human language-analyzing it to inform diagnoses and treatment recommendations-may introduce uncertainty due to the inherent subjectivity and variability of human communication. The vulnerability of NLP-based AI systems in accurately understanding natural language could pose risks to patient safety, potentially leading to misinformation, diagnostic errors, and violations of human rights^(50)^.

Based on the articles included in this scoping review, the primary strength of AI in PHC lies in its capacity for early diagnosis^(24,26,27,33,36,38,46)^. Early detection significantly impacts health outcomes, particularly for conditions with high mortality rates, thereby promoting the preservation of life and health^(25,26,28,29,32,35,36)^. Furthermore, AI enhances the efficiency of resource allocation through its diagnostic accuracy, enabling the formulation of appropriate treatment plans from the outset. This minimizes the risk of ineffective interventions that could compromise patient outcomes or even endanger lives^(23,24,26,30,36,46)^.

AI also contributes to knowledge generation within the healthcare sector by enabling the identification of trends and patterns that may otherwise go unnoticed. This facilitates the development of new insights and approaches to diagnosis and treatment, thereby strengthening the scientific foundations of PHC^(23)^. Additionally, AI can streamline administrative processes, saving time and improving decision-making efficiency. By automating routine tasks, AI allows healthcare professionals to concentrate on complex clinical responsibilities, enhancing overall care delivery.

From an economic perspective, investment in AI is seen as a long-term strategic decision. Although initial costs may be substantial, the potential for operational efficiency, improved care quality, and support for public health policy justifies the expenditure^(23)^. Another notable advantage is the ability of AI tools to generate easily interpretable results. This accessibility ensures that healthcare professionals can effectively utilize AI systems without requiring specialized expertise in data science or algorithmic interpretation^(25)^.

Moreover, AI has demonstrated potential in early risk stratification, thereby reducing mortality and contributing to increased life expectancy^(23,28,32,35,39,44,48)^. A high degree of concordance has been reported between AI-generated diagnoses and those made by clinical specialists^(25)^, reinforcing AI’s value as a complementary diagnostic tool.

However, several challenges in the effective implementation of AI in PHC were also identified. A major barrier is the limited availability of relevant variables and the development of accurate search algorithms, which hinders AI’s potential^(25,29,31,34,35,37)^. While AI may support continuous diagnosis, persistent issues such as restricted access to essential medications and insufficient healthcare personnel for treatment administration limit its practical impact. These gaps, coupled with organizational challenges within PHC centers, reflect broader systemic issues in the Brazilian healthcare system^(25,35,39,43,47)^.

Financial constraints, especially regarding the procurement of high-cost medications, further impede the effective deployment of AI technologies^(25,35,39,45)^. Additionally, disparities in access to PHC exacerbate health inequities, affecting the quality of life for individuals with both diagnosed and undiagnosed conditions. These systemic barriers must be addressed to ensure the equitable and effective integration of AI in PHC^(34)^.

Importantly, AI alone cannot overcome structural limitations such as unequal access to care and essential healthcare resources. Therefore, a key challenge lies in harnessing AI to reduce, rather than reinforce, existing health disparities. Another pressing issue is the lack of preparedness for emerging diseases, as seen in the global response to COVID-19. AI has the potential to serve as a responsive tool in such contexts, aiding the development of strategies to improve public health and quality of life^(37)^.

Limited internet connectivity, particularly in remote or underserved areas of Brazil, represents a significant barrier to the adoption of AI technologies in PHC. Without reliable digital infrastructure, the implementation of AI-based interventions remains constrained, undermining efforts to uphold the right to health^(33)^.

To move toward a more efficient, equitable healthcare system grounded in social justice, it is essential to address these structural and technological limitations. The selective application of AI in regions with the necessary infrastructure creates a digital divide, marginalizing populations without access to digital resources^(33,34,37)^. Therefore, the foundational shortcomings of healthcare systems must first be addressed. Only then can AI be effectively integrated to support progress toward the Sustainable Development Goals^(51)^. Addressing these issues is a critical step toward ensuring equitable access to PHC and realizing the universal, inalienable right to health^(52)^.

### Study limitations

Among the limitations of this review is the absence of a Google Scholar search or a manual search for the references in the included studies. This approach was not adopted, as we focused solely on including scientific literature in the review, and since the selected studies already provided valuable information.

### Contributions to the Field

This article helps us to contextualize ourselves in a social dynamic that faces stagnation in the face of the continuous advancement of technological reality. The disproportion of everyday situations prompts us to reflect on proposals that seek to establish a rational balance between the benefits and challenges of technology applied in society. The need for new technological alternatives and the violation of human rights, especially in the field of health, motivate us to delve deeper into the correct and effective applicability of technology that contributes to social equality.

## CONCLUSIONS

The use of AI in PHC represents a potentially significant advancement in reducing health disparities, owing to its versatility and broad range of applications. The integration of AI into PHC has demonstrated numerous strengths, particularly in its capacity to generate new knowledge in the health field, enhance service delivery, improve diagnostic accuracy, and facilitate early disease detection.

From a holistic perspective, the appropriate and ethical integration of AI into healthcare systems offers considerable societal benefits. Its development and application must, however, be guided by robust ethical and legal frameworks that uphold human rights. When focusing solely on its strengths, AI emerges as a valuable tool in Brazil’s PHC system, being able to support the state in fulfilling its constitutional obligation to guarantee the universal right to health. If implemented equitably across the country, AI has the potential to improve service delivery in PHC by reducing complications associated with diagnostic errors, mismanagement, and other systemic inefficiencies.

Nevertheless, this scoping review has identified several critical challenges to the equitable implementation of AI in PHC. These include limited data availability, inadequate internet connectivity, and restricted access to PHC services for segments of the population. Such barriers-clearly linked to broader issues in healthcare system management and public policy-pose significant obstacles to the effective and fair integration of AI. These infrastructural and systemic factors are essential preconditions for the successful deployment of AI technologies.

Although the potential benefits of AI in PHC are substantial, its implementation in Brazil’s current healthcare context would be insufficient to fully realize the right to health as a fundamental human right. Crucially, structural challenges, most notably, ensuring equitable access to healthcare for the entire population, which must be addressed as a priority. Without resolving these foundational issues, the integration of AI alone cannot fulfill the broader objective of achieving health equity and universal access.

## Data Availability

The research data are available within the article.
